# The role of head and hand movements for infants’ predictions of others’ actions

**DOI:** 10.1007/s00426-017-0939-6

**Published:** 2017-12-21

**Authors:** Benjamin Koch, Janny Stapel

**Affiliations:** Uppsala Child and Baby Lab, Department of Psychology, Uppsala University, Box 1225, 751 42 Uppsala, Sweden

## Abstract

In everyday life, both the head and the hand movements of another person reveal the other’s action target. However, studies on the development of action prediction have primarily included displays in which only hand and no head movements were visible. Given that infants acquire in their first year both the ability to follow other’s gaze and the ability to predict other’s reaching actions, the question is whether they rely mostly on the hand or the head when predicting other’s manual actions. The current study aimed to provide an answer to this question using a screen-based eye tracking setup. Thirteen-month-old infants observed a model transporting plastic rings from one side of the screen to the other side and place them on a pole. In randomized trials the model’s head was either visible or occluded. The dependent variable was gaze-arrival time, which indicated whether participants predicted the model’s action targets. Gaze-arrival times were not found to be different when the head was visible or rendered invisible. Furthermore, target looks that occurred after looks at the hand were found to be predictive, whereas target looks that occurred after looks at the head were reactive. In sum, the study shows that 13-month-olds are capable of predicting an individual’s action target based on the observed hand movements but not the head movements. The data suggest that earlier findings on infants’ action prediction in screen-based tasks in which often only the hands were visible may well generalize to real-life settings in which infants have visual access to the actor’s head.

## Introduction

Other people are a critical component of human life. For that reason, abilities underlying fluent social interaction and the understanding of others’ behavior are important. Consequently, much contemporary psychological research is dedicated to unraveling how action understanding develops early in life. Predictive looks, defined as looks to the (sub)goal of an observed action before that action is completed, are considered a hallmark of action understanding (Gredebäck & Falck-Ytter, 2015; Hunnius & Bekkering, 2014). Predictive looks are considered useful for action understanding because end points or turning points of actions frequently provide information relevant for understanding the purpose of the action: for drinking, one lifts a cup to the mouth, for clapping, the hands need to touch one another (Kilner, Friston, & Frith, 2007; Newtson, Engquist, & Bois, 1977; Uithol & Paulus, 2014). Hence, action prediction can form an important basis for action understanding, although action understanding requires more than prediction alone (Prinz, 2006). Many screen-based studies on action prediction have focused on infants’ ability to predict others’ goals based on observing the movements of the actor’s hand while leaving out the actor’s head (Ambrosini, Reddy, de Looper, Costantini, Lopez, & Sinigaglia, 2013; Elsner, Bakker, Rohlfing, & Gredebäck, 2014; Falck-Ytter, Gredebäck, & von Hofsten, 2006; Gredebäck & Kochukhova, 2010; Kanakogi & Itakura, 2011; Stapel, Hunnius, & Bekkering, 2015). The reason for leaving the actor’s head out of the stimulus videos is that the actor’s head movements and gaze turns are known to trigger gaze shifts in the infant from the actor to the object the actor looks at, a phenomenon called gaze-following (Johnson, Slaughter, & Carey, 1998). Although this approach of studying action prediction in isolation helps to learn to appreciate the role of body movements, it leaves the question of how infants predict actions in real-life situations unanswered (Rosander & von Hofsten, 2011, provided evidence for infants’ ability to predict real world actions). The current study aims to start bridging the gap between live studies (which typically display the actor’s head) and screen-based action prediction studies (which typically do not display the actor’s head) by comparing in a screen-based study infants’ predictions of an actor’s action when the actor’s head is visible or invisible.

The work of Flanagan and Johansson (2003) has formed an important source of inspiration for developmental studies on action prediction. Their adult participants displayed remarkably similar patterns of eye–hand coordination when stacking blocks themselves and when observing the experimenter stacking blocks. It appeared almost as if the eyes of the observers guided the hands of the experimenter, as the observers’ gaze landed on the location where the experimenter’s hand would go ahead of the actual hand action. These predictive looks (gaze preceding the hand) were taken as an indication that observers run the same computations based on the same action plans when observing an action as when performing the action themselves. In Flanagan and Johansson’s study, participants only saw the hands of the experimenter and not his face. This setup allowed the researchers to rule out the possibility that observers coordinated their own gaze with the experimenter’s gaze rather than with his hands when observing the block-stacking actions.

Developmental studies examining predictions of others’ actions have displayed actors performing manual actions without the actor’s face in view (Ambrosini et al., 2013; Elsner et al., 2014; Falck-Ytter et al., 2006; Gredebäck & Kochukhova, 2010; Kanakogi & Itakura, 2011; Stapel et al., 2015), following Flanagan and Johansson (2003). Results of these studies demonstrated that infants are indeed capable of predicting others’ actions, which shows from the infants’ predictive looks at the target of an action prior to action completion (Falck-Ytter et al., 2006; Hunnius & Bekkering, 2010; Kanakogi & Itakura, 2011; Stapel et al., 2015).

Studies on developmental action prediction elucidate, at least partially, the potential mechanisms underlying action prediction. Own motor ability, and thereby motor development, seems crucial for predicting others’ actions (Ambrosini et al., 2013; Elsner et al., 2014; Falck-Ytter et al., 2006; Gredebäck & Kochukhova, 2010; Kanakogi & Itakura, 2011; Stapel et al., 2015). This is in line with the mirror neuron or motor account of action prediction, which postulates that similar (sensorimotor) brain areas are recruited when acting and when perceiving actions of others (Prinz, 2006; Wilson & Knoblich, 2005; Wolpert, Doya, & Kawato, 2003). Some scholars have suggested that the mirror system is readily functioning at birth (Craighero, Leo, Umiltà, & Simion, 2011; Lepage & Théoret, 2007), which implies that infants should be capable of predicting actions regardless of their own motor repertoire. However, action prediction does not need to rely on motor processes, as visual experience can provide an alternative route to action prediction. Through statistical or associative learning, infants may learn that certain bodily movements, such as reaching, are often followed by certain end effects, such as holding an object in hand. The formed action–effect associations may then in turn be used to predict the action end effect when perceiving the action again (Elsner & Hommel, 2001; Paulus, van Dam, Hunnius, Lindemann, & Bekkering, 2011). Data of Hunnius and Bekkering (2010) illustrate that infants can indeed predict actions based on solely observational experience. In their study, 6-month-old infants made predictive eye movements towards the ear of an actor. Predictive gaze to the ear more frequently occurred when the actor brought a phone to her ear than when she brought a cup to her ear. Most likely, these predictive eye movements sprang from seeing others bringing phones to their ear, as infants of that age are not yet motorically capable of bringing objects to their ear themselves. Note that in the study by Hunnius and Bekkering, the actor did not move her head or eyes in a way that could reveal where the movement would end.

Apart from visual experience and motor processes, predictions of others’ actions may also rely on detecting direction cues in the stimuli, or on processing of socially relevant information. These latter two explanations are frequently mentioned in the gaze-following literature. Gaze direction, sometimes inferred from head orientation but most explicitly signaled by the eye direction, provides socially relevant information by revealing where the other agent is attending to (Driver et al., 1999; Langton & Bruce, 1999). That is, any given space, be it a room inside a house or a marketplace outside, contains an abundance of visual information that cannot be processed in detail all at once (Desimone & Duncan, 1995). Therefore, it is useful to look and see what the other intentional agent is attending to. The other agent might have selected a certain object of attention for a good reason, and it may prove to be a relevant object of attention for us as observers too (Triesch, Teuscher, Deák, & Carlson, 2006). This explanation of why we follow gaze assumes the ability to attribute intention to other agents, and to use that attribution to follow the gaze of the agent. Whether infants have the capacity to attribute intentionality (Gergely, Nádasdy, Csibra, & Bíró, 1995; Luo & Baillargeon, 2005; Sirois & Jackson, 2007) and productively use intention attributions to follow gaze is disputed (Butterworth & Jarrett, 1991; Johnson et al., 1998). Scholars favoring a more lenient view postulate that infants deduce from the social partner’s gaze direction where the person is looking at, without attributing intentionality to that partner. They may learn from experience that looking in the same direction as another person often leads to seeing interesting objects or events (Butterworth & Jarrett, 1991), which may through reward-based learning lead them to exhibit the same (gaze-following) behavior in subsequent situations.

Despite the controversy around the mechanisms underlying gaze following, it is undisputed that infants develop the capacity to follow gaze. The earliest report described that 30% of the tested 2-month-olds followed gaze, gradually increasing over age to 100% at 14 months (Scaife & Bruner, 1975). More complex gaze-following, such as following the experimenter’s gaze to an object located behind oneself and distinguishing smaller gaze angle differences, emerges in the second year of life (Butterworth & Jarrett, 1991). Both head and eye movements of the experimenter can independently elicit gaze-following behavior, but both cues combined result in the highest gaze-following scores (Butterworth & Jarrett, 1991; Corkum & Moore, 1995).

At first sight, it might seem that action prediction relies on different mechanisms when it is cued by head movements or hand movements, respectively. It seems a common intuition amongst scholars that predictions based on head and eye movements spring from mechanisms for social understanding and directional cueing, whereas predictions based on hand movements spring from sensorimotor mechanisms such as motor simulation or action–effect associations. However, head and eye movements not only convey directional and socially relevant cues which might allow intention attribution, head and eye movements also could potentially be simulated by the motor system. Furthermore, head and eye movements may also have formed the basis for action–effect associations (Hommel, Müsseler, Aschersleben, & Prinz, 2001) acquired through learning from observation (see for evidence on the acquisition of action–effect associations through observational learning: Paulus et al., 2011). Likewise, there is no strong theoretical reason why goal-directed hand movements could not bring about predictive eye movements through attributing intentions to the observed actor, if one would want to assume that infants have the capacity to attribute intentions. As such, all four explanations mentioned—observational learning, motor simulation, intention attribution, and detecting directional signals—count as viable explanations for both hand- and head-based action prediction. Though the current study was hence not designed to disentangle the mechanism underlying action prediction based on hand movements or on head movements, it may still yield insights relevant for the debate on these mechanisms.

Recent live-interaction studies provide some first indications that infants prioritize hand over head movements as joint attention between infants (ranging between 11- and 24-months of age) and their parents were more frequently established through attending to each others’ hands and hand-held objects than by means of gaze-following (Yu & Smith, 2013, 2017). These studies suggest that although infants are capable of following gaze and might use head turns as cues for predicting the subsequent action, they might rely more on hand than on head movements when predicting others’ actions.

The current study aimed to test the relative contributions of head and hand movements for infants’ action prediction. To that end, 13-month-old participants were presented with video clips of a model stacking rings to a pole while the participants’ gaze was tracked. In half of the videos, the model’s head was visible. In the other half of the videos, her head was invisible because a black rectangle occluded her head and neck. Looking time analyses were used to describe potential differences in duration of looks to the critical aspects of the scene, namely the model’s hand, her head, and the target location of the action. Due to infants’ preference for faces, we expected participants to look longer at the region in the scene where the head was displayed when it was visible compared to when the head was occluded. As a consequence of looking at the head during the action, infants were expected to look shorter at the model’s hand and the action target when the actress’ head was visible. In addition to looking time, gaze-arrival time was calculated, which is the difference in time between arrival of the model’s hand and participant’s gaze at the target. Positive gaze-arrival times indicated predictive gaze as the eyes precede the hand, whereas negative gaze-arrival times signaled reactive gaze. As the model portrayed natural actions, her head always started turning towards the target before movement onset of the hand. Hence, if infants made use of cues from head orientation, the gaze might be more ahead of the action when the head was visible compared to when it was not visible. Alternatively, infants might focus their attention on the model’s head when possible, at the expense of predicting the target. A face provides more than movement information only; it also carries other socially relevant information such as emotions, gender, and identity (Haan & Nelson, 1997; Quinn, Yahr, Kuhn, Slater, & Pascalis, 2002; Serrano, Iglesias, & Loeches, 1995). Processing this additional information might be prioritized over predicting the ongoing action and hence lead to later gaze-arrival. In addition to the conditional contrasts, exploratory analyses were conducted to obtain a description of gaze behavior within the head-visible condition. It was deemed of interest to explore whether infants would display more looks to the target following looks at the head or looks at the hand. If target looks would often follow from looks at the head, then the question is whether gaze landed earlier at the target following either a head or a hand look. As the head turn was the first sign of the impending action, infants might be quicker to look at the target if they focused on the head rather than on the hand. Lastly, to assess whether such a dichotomy of hand- versus head-based target looks was justified, we counted the number of trials in which the infants triangulated by looking at the hand as well as the head before looking at the target.

## Methods

### Participants

Twenty-two participants (15 girls) took part in the experiment. The participating infants were 13 months old (*M* = 13.1 months, SD = 0.2). Three other infants visited the lab but did not contribute data to the final analyses due to fussiness (*n* = 1), poor eye tracking data (*n* = 1; exclusion criterion: more than 50% missing samples) or parental interference (*n* = 1). Infants were recruited from a database comprising infants whose parents had indicated earlier to be interested in participating in research with their child. Infants were primarily from European white middle-class backgrounds. Parents signed an informed consent form prior to commencing the study. Participants and their parents were compensated for the lab visit with a gift card (approx. 10 Euros) for a local bookstore.

### Materials

The stimulus materials were short videos without sound (1280 × 1024 pixels; 29.2 s; frame rate 25 fps) displaying an actor seated behind a table. A pile of four colorful rings was positioned at the right-hand side of the actor, and a post at the left-hand side. The video started with a still frame in which the actor faced the child but looked downwards. After half a second, the actor turned her head towards the post and subsequently reached for and grasped the base. The actor then turned her head towards the pile and started stacking the rings one by one on the post. The video included four reaching-to-grasp-a-ring actions (duration: *M* = 1.49 s, SD = 0.31), and four transporting-to-place-a-ring actions (duration: *M* = 1.70 s, SD = 0.20). The head movement always preceded the hand movements, which is natural as hand movements normally lag behind eye movements in skilled eye–hand coordination (Flanagan & Johansson, 2003; Sailer, Flanagan, & Johansson, 2005). The difference between the onset of head and hand movement was on average 0.43 s (SD = 0.31) and 0.33 s (SD = 0.29) for the transporting and reaching actions, respectively. A second version of this video was created by placing a black rectangle over the upper part of the video (1280 × 497 pixels) which fully occluded the head and neck of the actor during the entire sequence of actions. To ensure some variation, horizontally flipped versions (i.e., left and right were switched) of these two distinct videos were made as well.

The videos were displayed at a Tobii T120 eye tracker using Tobii Studio software (Tobii AB, Sweden). Infants’ gaze was recorded at a sampling rate of 60 Hz. Tobii’s I-VT filter was used to extract fixations from the raw data.

**Fig. 1:**
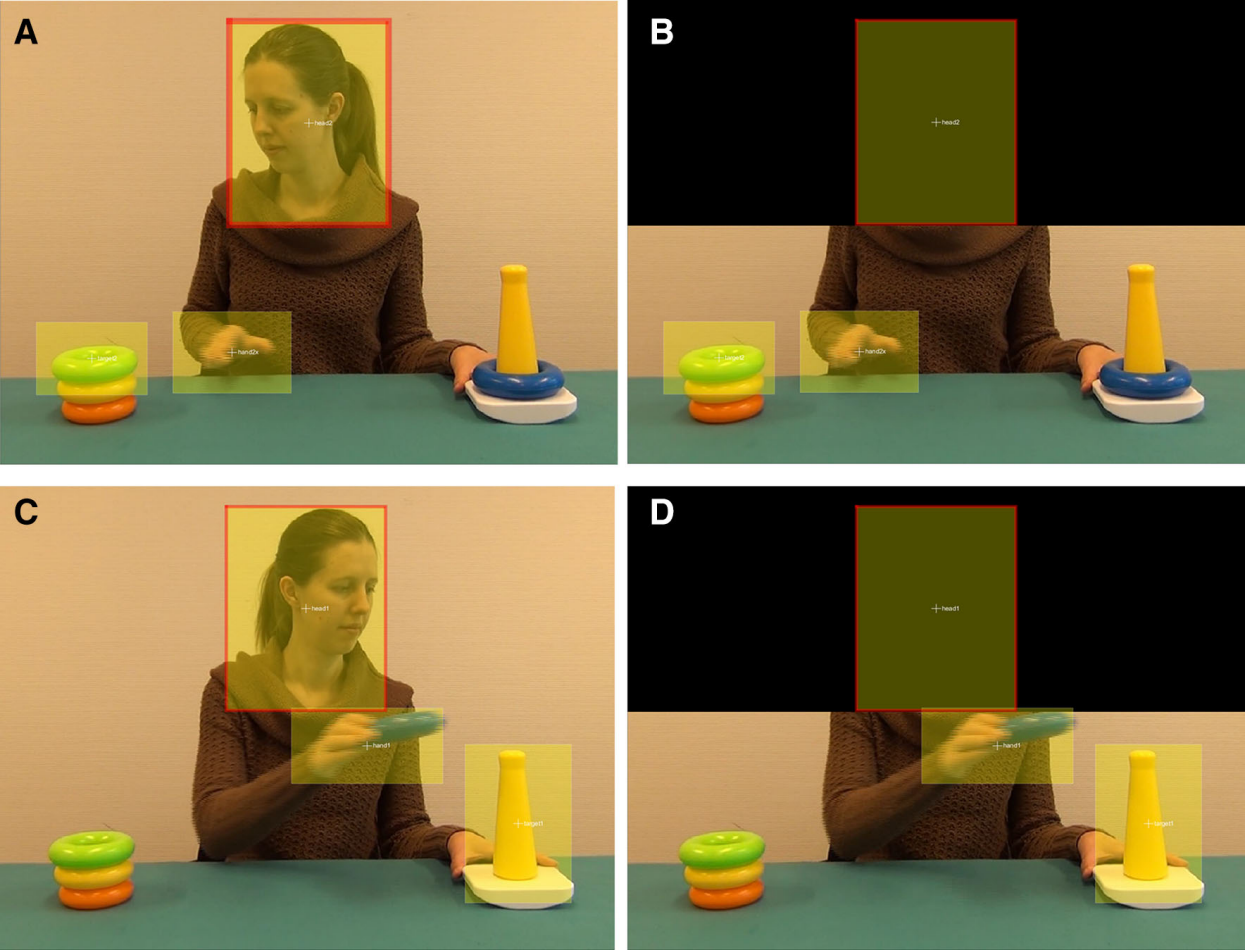
Example frame from the stimulus videos with the head-visible (HV condition) and the head-invisible (HI condition) for the Reaching action (HV: **a**, HI: **b**) and for the Transporting action (HV: **c**, HI: **d**)

### Procedure

Upon arrival at the lab, parents received a short introduction to the study and were asked not to talk to their child during the test. A five-point calibration procedure was administered in which contracting and expanding circles were presented in each corner and in the middle of the screen. The calibration clips were accompanied by sounds to attract the infant’s attention to the screen. After successful calibration the test was started. The test consisted of three videos for the head-visible (HV) condition and three videos for the head-invisible (HI) condition. Hence, infants observed twelve reaching-followed-by-transporting actions per condition (three videos × four reaching-followed-by-transporting actions per video). A within-subjects design was applied, meaning that all infants observed the videos of both conditions. After every other video, a short attractive audiovisual clip was presented to maintain the infant’s attention to the screen. Two random sequences were generated and in alternating fashion, participating infants were either assigned to the first or the second random sequence of the six stimulus videos. The complete test took a little more than 3 min in total. Parents were shown a gaze replay of the videos and received an explanation of the study’s purposes. The infants shortly received the opportunity to stack the tower they had seen in the videos themselves.

### Data reduction and analysis

A Matlab-based workflow tool called TimeStudio (Nyström, Falck-Ytter, & Gredebäck, 2016) was used for the gaze data analyses. The stream of actions was parsed into eight different actions: four transporting and four reaching actions. For both action types, a target area of interest (AoI) was defined around the end point of the action. The target AoI of the transporting action covered the post and was 220 pixels wide and 350 pixels high. The target AoI of the reaching action spanned the ring that would be grasped next and was 230 × 160 pixels. The target AoIs did not overlap with the black rectangle of the HI condition. For both action types, a head AoI (330 × 450 pixels) was defined around the head of the actor, with the head in the center (Fig. 1). A hand AoI was defined to cover the hand or, in case of the transporting action, the hand and the hand-held ring. The edge of the hand AoI in some cases shortly overlapped with the black rectangle, as the spatial resolution of this type of data asks for AoIs that are slightly larger than the actual object of interest. The hand AoI was a moving AoI which expanded and contracted in size during the course of the actions, reflecting changes in the size of the image of the hand in each frame of the video: when crossing midline, the hand was viewed frontally and hence the image tended to be smaller than when at the starting point, where the hand was viewed from a more sideways perspective. Size and location of the hand AoI was determined for a number of key frames, after which Timestudio applied linear transformations to its size and its location to render a moving AoI. The size of the hand AoI was approximately 300 × 200 pixels.

All trials for which gaze data was recorded, including the first trial, were included in the analyses. Only specific time windows of the videos were included in the analyses. The analysis time windows started at action onset. Action onset was signaled by the start of the head movement, regardless of whether the head was visible or rendered invisible to ensure comparability between conditions. The analysis time windows ended 1 s after the model’s hand had entered the target AoI. Fixations occurring later than 1 s after the hand had entered the target AoI were thus discarded as these fixations were not thought to be based on the observed actions.

Looking times and the numbers of trials per condition were assessed to verify whether infants observed the same number of actions per condition, and whether the time spent looking at the actions was comparable between the HI and HV conditions. In case of an unequal distribution of trials over conditions, the interpretation of conditional differences becomes non-trivial. The number of trials was counted in which the participant displayed a fixation to the head, hand, or target AoI during the same time windows that were used for the main analyses.

A number of indices and comparisons were considered of interest in addressing the main question. First of all, it was deemed of interest to describe the relative looking durations to the respective AoIs, to verify whether the infants looked at the head, and whether the visibility of the head led to shorter looking times to the target and the acting hand. The relative looking time to the separate AoIs was expressed as a percentage of the total time the infant looked at the screen during that action time interval.

The main analyses focused on the gaze-arrival time at the target: the difference in time was calculated between the arrival of the model’s hand at the target AoI and the moment of the first look to the target AoI—after having looked at the hand. Thus, a target fixation had to meet four criteria to be used in the gaze-arrival time analyses: (1) should land in the target AoI, (2) should land before the end of the analysis time window (closing 1 s after the model’s hand entered the target AoI), (3) should be preceded by a fixation to the hand AoI, (4) the fixation to the hand AoI had to take place after action onset (start of head turn). When gaze arrived at the target before the hand did, the difference in time between hand arrival and gaze arrival was positive. These positive gaze-arrival times thus indicated predictive looking behavior, whereas negative gaze-arrival times represented following the action or gaze lagging behind the displayed action.

Lastly, we conducted exploratory analyses on the gaze behavior in the HV condition. We aimed to find whether infants would base their predictions on the head movements rather than the hand movements whenever the head was visible, and hence looked at the target after having looked at the head, or made hand-based predictions in these cases as well. The number of trials in which a target look followed after a look to the hand was compared to the number of trials in which a target look followed after a look to the head. In case the number of trials with hand-based target looks was not different from head-based target looks, follow-up analyses were conducted comparing the gaze-arrival times. For these analyses, trials in which the head was visible were first divided into four categories: the infant has looked at the target after having looked at both the head and the hand (1), after having looked at only the hand (2), only the head (3), and remaining trials (4). In all analyses, gaze-arrival times or looking durations were averaged per condition. Statistical comparisons were made in IBM SPSS Statistics 22 (IBM, NY, USA). Analyses were conducted stepwise to restrict the number of analyses and to focus the analyses on the questions emerging from the design and where necessary on questions emerging from the data. Alpha-level correction was not applied.

## Results

### Number of observed trials and looking times per condition

Paired-samples *t* tests revealed that the number of attended trials was not different for the HV and the HI condition, neither during the reaching actions (*M*_HV_ = 10.9 trials, SD_HV_ = 1.2; *M*_HI_ = 11.2 trials, SD_HI_ = 1.2), *t*(21) = 0.32, *p* = 0.246, nor during the transporting actions (*M*_HV_ = 11.0 trials, SD_HV_ = 1.2; *M*_HI_ = 11.0 trials, SD_HI_ = 1.2), *t*(21) = 0.05, *p* = 0.866.

Paired-samples *t* tests comparing the looking times indicated that the time spent looking at the screen was neither different for the HV and the HI condition for the reaching action (*M*_HV_ = 1927 ms, SD_HV_ = 309, *M*_HI_ = 1917 ms, SD_HI_ = 337), *t*(21) = 0.28, *p* = 0.785, nor for the transporting action (*M*_HV_ = 2105 ms, SD_HV_ = 305, *M*_HI_ = 1990 ms, SD_HI_ = 398), *t*(21) = 1.56, *p* = 0.135.

### Head-visible vs. invisible: looks to hand, head, and target

#### Reaching actions

Infants were found to look at the head AoI during the reaching actions when the head was visible and hardly ever when the head was invisible (*M*_HV_ = 27.0%, SD_HV_ = 12.9, *M*_HI_ = 0.2%, SD_HI_ = 0.7), *t*(21) = 9.63, *p* < 0.001. Looking at the head did not occur at the expense of the looking duration to the target AoI: participants looked to the target around 40% of the time in both conditions (*M*_HV_ = 43.1%, SD_HV_ = 11.8; *M*_HI_ = 47.2%, SD_HI_ = 11.1), *t*(21) = 1.57, *p* = 0.131. When infants had the opportunity to look at the head, they looked shorter at the hand (*M*_HV_ = 11.5%, SD_HV_ = 6.1; *M*_HI_ = 15.7%, SD_HI_ = 6.0), *t*(21) = 2.89, *p* = 0.009.

#### Transporting actions

**Fig. 2:**
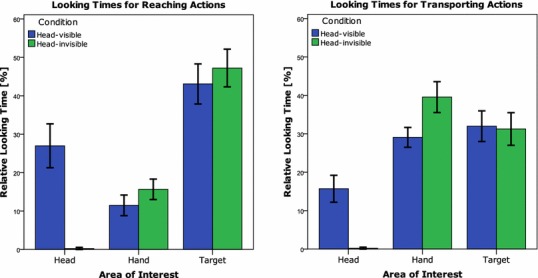
Mean relative looking times at the Areas of Interest (Head, Hand, Target) per condition for the Reaching actions (**a**) and Transporting (**b**) actions. The error bars represent 95% confidence intervals

Participants tended to look at the head AoI when the head was visible (*M* = 15.7%, SD = 7.9) during the transporting action and hardly ever when the head was invisible (*M* = 0.2%, SD = 0.6), *t*(21) = 9.22, *p* < 0.001. However, the duration of looks to the target AoI during the transporting action was not found to be different when the head was visible (*M* = 32.0%, SD = 9.0) or invisible (*M* = 31.3%, SD = 9.6), *t*(21) = 0.43, *p* = 0.674. Infants were found to look longer at the hand and handheld ring when the head was occluded (*M* = 39.6%, SD = 9.1) compared to when the head was visible (*M* = 29.1%, SD = 5.8), *t*(21) = 4.59, *p* < 0.001 (Fig. 2).

### Head-visible vs. invisible: effect on hand-based predictions

Infants might predict the target based on the hand movements both when the actor’s head is visible and when the head is invisible. For both action types, paired-samples *t* tests were employed to investigate whether the time of gaze-arrival at the target differed between conditions.

#### Reaching actions

For the reaching action, no differences were found in the gaze-arrival times between the HV and HI conditions, *t*(21) = 0.41, *p* = 0.685. Infants showed predictive looks to the target for the reaching action, as the gaze-arrival times were significantly above zero, for both the HV (*M*_HV_ = 185 ms; SD_HV_ = 359), *t*(21) = 2.41, *p* = 0.025, as well as the HI condition (*M*_HI_ = 146 ms; SD_HI_ = 288), *t*(21) = 2.37, *p* = 0.028.

#### Transporting actions

**Fig. 3:**
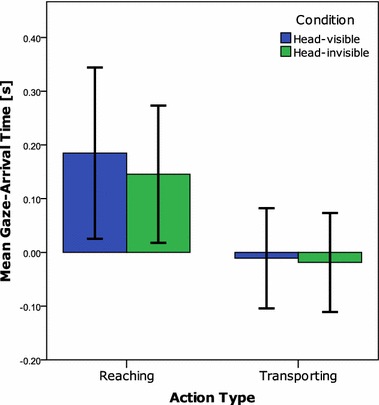
Mean gaze-arrival times at the target for the Reaching actions and Transporting actions split by condition. The error bars represent 95% confidence intervals

Results for the transporting action revealed no difference in gaze-arrival times between HV and HI, *t*(21) = 0.17, *p* = 0.866. Moreover, the gaze-arrival times indicated that infants followed the transporting action rather than predicted the target of the transporting action (*M*_HV_ = − 11 ms, SD_HV_ = 210; *M*_HI_ = − 19 ms, SD_HI_ = 208). One-sample *t* tests confirmed that the gaze-arrival times were not different from zero, HV: *t*(21) = 0.24, *p* = 0.810; HI: *t*(21) = 0.42, *p* = 0.678, but significantly shorter than 200 ms, HV: *t*(21) = 4.22, *p* < 0.001; HI: *t*(21) = 4.09, *p* = 0.001, which suggests that the infants did follow the action (Fig. 3).

### Head-visible: effectiveness of hand- vs. head-based predictions

Table 1 provides an overview of the number of trials falling into the four different categories of target looks.

**Table 1:** Mean number of trials listed per category and type of action

	Reaching actions	Transporting actions
Category 1: head and hand	2.1 (1.7)	3.0 (1.5)
Category 2: hand only	3.2 (2.5)	5.3 (2.4)
Category 3: head only	3.0 (1.8)	0.4 (0.5)
Category 4: remaining trials	3.7 (2.4)	3.4 (1.9)

Standard deviations are reported within brackets

#### Reaching actions

For the reaching action, the frequency of target looks following head looks (category 3, *M* = 3.0 trials, SD = 1.8) was not found to be different from the frequency of target looks following hand looks (category 2, *M* = 3.2, SD = 2.5), *t*(21) = 0.30, *p* = 0.767. However, the time of gaze-arrival at the target was clearly earlier after looking at the hand (*M* = 546 ms, SD = 393) compared to after looking at the head (*M* = − 235 ms, SD = 293), *t*(15) = 8.21, *p* < 0.001. Moreover, whereas hand-based looks to target were predictive, one-sample *t* test against zero: *t*(17) = 5.62, *p* < 0.001, head-based looks were reactive, *t*(19) = 2.59, *p* = 0.018. Relatively frequently, infants looked at both the hand and the head in one trial and looked at the target after these fixations as well (category 1, *M* = 2.1 trials, SD = 1.7). In all but three cases, this involved a look to hand and head followed by target fixation rather than a more complex pattern such as hand–target–head–target or head–target–hand–target. Infants displayed reactive gaze in trials in which they looked at both head and hand before looking at the target (*M* = − 235 ms, SD = 265), *t*(17) = 3.77, *p* = 0.002.

#### Transporting actions

**Fig. 4:**
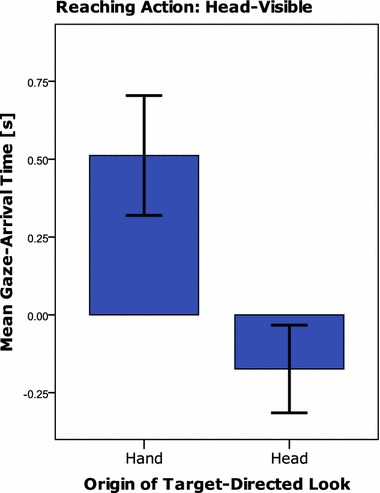
Mean gaze-arrival times at the target for the Reaching action in the head-visible condition. Target looks either followed a look at the hand (left bar) or a look at the head (right bar). The error bars represent 95% confidence intervals

Infants looked more frequently at the target of the transporting action in response to a look at the hand and not the head (category 2, *M* = 5.3 trials, SD = 2.4) than in response to a look at the head and not at the hand (category 3, *M* = 0.4 trials, SD = 0.5), *t*(21) = 8.86, *p* < 0.001. Given the low frequency of purely head-based target looks, it seemed not sensible to compare the gaze-arrival times of these target looks between these situations. Infants relatively frequently looked at both the hand and the head before looking at the target (*M* = 3.0, SD = 1.5), although this occurred less frequently than looks to the target after a look to the hand but not the head, *t*(21) = 3.17, *p* = 0.005. Nearly all trials in which infants looked at both hand and head consisted of hand–head–target or head–hand–target trajectories rather than more complex scanning patterns (such as hand–target–head–target or head–target–hand–target). Looking at the head seems, however, detrimental for the gaze-arrival time, as target fixations occurred earlier if the infant had only looked at the hand prior to looking at the target (category 2, *M* = 89 ms, SD = 353) compared to having looked at both hand and head prior to a target look (category 1, *M* = − 156 ms, SD = 262), *t*(20) = 2.47, *p* = 0.022 (Fig. 4).

## Discussion

The current study aimed to investigate whether infants’ predictions of target-directed actions are affected by the visibility of the actor’s target-directed head movements. To that end, 13-month-old infants observed videos of a model reaching and grasping rings, transporting and stacking them on a pole. Analyses of the infants’ gaze showed that when the head was visible, infants looked at the model’s head and looked shorter at the hand performing the action as compared to when the head was invisible. During the transporting phases, infants followed rather than predicted the action. Infants predicted the targets of the reaching phases. Critically, gaze-arrival times at the target were not found to be different for when the model’s head was visible or invisible, neither during the transporting nor during the reaching phases. Interestingly, when the head was visible, looks to target arrived later if the infants focused on the head of the model instead of (solely) on her moving hand.

The infants displayed predictive gaze for the reaching actions. In contrast, the infants merely followed the transporting action. These results mimic two other studies (Gredebäck, Stasiewicz, Falck-Ytter, von Hofsten, & Rosander, 2009; Sciutti, Lohan, Gredebäck, Koch, & Rohlfing, 2016) reporting predictive gaze in 14-month-olds for reaching but not transporting actions. Potentially, infants preferred to look at the object that was being transported rather than at the target because the object was colorful and moving (Dannemiller, 2005).

More important for the central question of the current study are the differences between the head-visible and the head-invisible conditions. As expected, participants looked at the model’s head when it was visible and hardly looked to the same region when the head was occluded. Faces convey many types of socially relevant information such as gender, identity, and emotion (Haan & Nelson, 1997; Quinn et al., 2002; Serrano et al., 1995). Infants are frequently confronted with faces from early on and orient already as newborns towards faces or face-like stimuli (Goren, Sarty, & Wu, 1975). They are already capable of discriminating basic facial emotional expression at 3 months of age (Serrano et al., 1995; Young-Browne, Rosenfeld, & Horowitz, 1977). More importantly, between 3 and 9 months infants gradually start to focus on faces which are embedded in complex, dynamical scenes (Frank, Vul, & Johnson, 2009). The current results are in line with these prior findings, as the participants looked at the model’s head at the expense of looking at the model’s hand when her head was visible.

The finding that infants looked less at the model’s hand when the head was visible was in line with the hypothesis. Interestingly, one would expect that shorter looking at the hand may also affect the predictive eye movements. Two competing hypotheses were formulated which described the potential effect of infants’ shifted attention towards the model’s face. It could either have led to earlier looks to the target because the head moved prior to the hand, or, alternatively, to later target looks because looks to the face could take critical processing time. Overall, the gaze-arrival time data showed no loss due to the visibility of the face as the gaze-arrival time of looks to the target were not found to be different for the head-visible and invisible conditions, an observation which held for both the transporting actions and the reaching actions. The second implication of these results is that infants did not benefit from the visibility of the head. Importantly, the findings suggest that prior results on infants’ action prediction in screen-based tasks (Ambrosini et al., 2013; Elsner et al., 2014; Falck-Ytter et al., 2006; Gredebäck, & Kochukhova, 2010; Kanakogi, & Itakura, 2011; Stapel et al., 2015) may very well hold in real-life settings as well. Our results are furthermore in line with recent findings (Yu & Smith, 2017) that illustrate that infants coordinate joint attention with their parents based on hand movements rather than through gaze-following.

A maybe even more important implication flowing from our findings is that infants do not predict others’ action outcomes through gaze-following, despite being capable of gaze-following at this age (Butterworth & Jarrett, 1991; Scaife & Bruner, 1975), but through observing other’s hand movements. During the transporting actions, target looks were hardly ever preceded by looks at only the face and not the hand, but were frequently preceded by looks at the hand and not the face. During reaching actions, the frequency of looks to target after a look to the head and not the hand was not found to be different from the frequency of target looks after a look to the hand and not the head. In other words, goal-directed eye movements could be based on either head or hand movements. However, first looking at the face during the reaching actions led to reactive target looks whereas first looking at the hand led to predictive target looks. Similarly, when infants looked at the model’s head and hand prior to looking at the target, the looks to the target arrived later in comparison to when target looks occurred after only a look to the hand. This held for both the transporting actions and the reaching actions. Together, the results indicate that infants’ target looks may spring from gaze-following, but are quicker when based on observing the actor’s hand instead.

The finding that action prediction seems to be based on observation of hand movements rather than head movements is congruent with the motor account or mirror system account of action prediction (Prinz, 2006; Wilson & Knoblich, 2005; Wolpert et al., 2003). Subsets of mirror neurons in the macaque brain were found to increase in firing rate during observation of some specific but not merely all manual actions (Rizzolatti, Fadiga, Gallese, & Fogassi, 1996). This action-specific mirror system activity is thought to reflect action simulation of not-yet-finished movements, generating predictions of the ongoing action (Kilner et al., 2007). Hence, according to these views, observing hand movements can be sufficient to predict the action and the head movement can thus safely be ignored. This is in line with the behavior displayed by the infant participants here: looking at both hand and head before looking at the target hardly occurred, and in cases infants did look at both hand and head, gaze arrived later at the target than in purely hand-based target looks. From a strong motor perspective (e.g., Gredebäck & Falck-Ytter, 2015), 13-month-olds should be capable of predicting both the transporting and reaching actions as they at least have a minimal capacity to perform both actions, which was not found in the current study.

The current data allows for and does not rule out alternative explanations for the mechanisms underlying action prediction. The study did not include explicit manipulations of potentially perceived intentionality (Johnson et al., 1998), direction cues (Butterworth & Jarrett, 1991), observational experience (Hunnius & Bekkering, 2010) or tests of motor processes (Elsner, D’Ausilio, Gredebäck, Falck-Ytter, & Fadiga, 2013; Kanakogi & Itakura, 2011) that could help disentangle which mechanism best explains developmental action prediction. Prior studies on action prediction in infancy provide strong evidence that both observational experience (Henrichs, Elsner, Elsner, Wilkinson, & Gredebäck, 2014; Hunnius & Bekkering, 2010) and motor processes (Ambrosini et al., 2013; Elsner et al., 2014; Falck-Ytter et al., 2006; Gredebäck & Kochukhova, 2010; Kanakogi & Itakura, 2011; Stapel et al., 2015) play a role in infants’ predictions of others’ actions (see also Hunnius & Bekkering, 2014).

In conclusion, the current study demonstrated that 13-month-olds’ gaze-arrival time at another’s action target was not different for a situation in which the other’s head was visible or invisible for the observing infant. This suggests that results from prior screen-based studies on action prediction in infants, in which the model’s face was not presented (Ambrosini et al., 2013; Elsner et al., 2014; Falck-Ytter et al., 2006; Kanakogi & Itakura, 2011; Stapel et al., 2015), are likely to generalize to real-world settings in which infants do have visual access to the actor’s head. While both gaze- and hand-following was observed, only looks to the hand led to predictive target-looks. In sum, 13-month-old infants were found to predict other’s reaching actions based on the hand but not the head movements of the other person.
